# P4HA2 contributes to cervical cancer progression via inducing epithelial-mesenchymal transition

**DOI:** 10.7150/jca.38401

**Published:** 2020-02-21

**Authors:** Yuan Cao, Qicai Han, Juan Li, Yanyan Jia, Ruitao Zhang, Huirong Shi

**Affiliations:** 1 Department of Gynaecology, the First Affiliated Hospital of Zhengzhou University, Zhengzhou 450014, China; 2 Key Laboratory of Clinical Medicine, the First Affiliated Hospital of Zhengzhou University, Zhengzhou 450052, China

**Keywords:** P4HA2, prognosis, metastasis, EMT, cervical cancer

## Abstract

**Background:** Cervical cancer is one of the most common gynaecological malignancies. Emerging studies have documented that prolyl-4-hydroxylase α subunit 2 (P4HA2) is involved in multiple processes of cancer progression. However, the functional roles of P4HA2 in cervical cancer progression remain to be elucidated.

**Methods:** P4HA2 mRNA and protein levels were examined in cervical cancer tissues and cell line by qRT-PCR and western blot. The correlation of the P4HA2 expression levels and prognosis of cervical cancer patients were analysed in TCGA cervical cancer cohort and tissue microarray (TMA) cohort. P4HA2 was silenced to evaluate its function on cervical cancer progression both *in vitro* and *in vivo*. Bioinformatics analysis was performed to investigate the underlying regulation mechanism of cervical cancer by P4HA2.

**Results:** We found that P4HA2 are markedly upregulated in cervical cancer tissues in comparison with adjacent non-neoplastic tissues. In addition, upregulation of P4HA2 was associated with shorter overall survival (OS) and relapse-free survival (RFS). Functionally, we demonstrated that P4HA2 knockdown attenuated cell proliferation, migration and invasion of cervical cancer cells. Furthermore, xenograft tumor mouse model experiment showed silencing P4HA2 significantly inhibited tumor growth *in vivo*. Mechanistically, bioinformatics analysis revealed that epithelial-mesenchymal transition (EMT) was involved in cervical cancer progression regulated by P4HA2 and we further confirmed knockdown P4HA2 suppressed the EMT process.

**Conclusion:** our results suggest that P4HA2 functions as an oncogene in promoting cervical cancer cell proliferation, migration and invasion by inducing EMT, which might be a promising prognostic factor and therapeutic target for cervical cancer.

## Background

Cervical carcinoma is a common aggressive malignancy and the fourth leading cause of cancer-related deaths among females worldwide, especially in China 1, 2. Despite significant progress in therapeutic strategies in the past few decades, the prognosis of cervical carcinoma remains poor, especially in patients with advanced stage disease 3. Distant metastasis remains the predominant mode of treatment failure and high mortality of advanced patients due to the highly invasive and metastatic potential of cervical cancer^4^. Though intensive studies focus on the tumorigenesis of cervical carcinoma, the underlying molecular mechanisms and efficient treatment strategies remain unclear.

Collagen biosynthesis and deposition involve multiple complex processes that are regulated by several post-transcription modification proteins, such as collagen prolyl-4-hydroxylase^5^. There are three subtypes of collagen prolyl-4-hydroxylases α isoforms (P4HA1, P4HA2 and P4HA3)^6^. P4HA2 has been reported to induce extracellular matrix remodelling under hypoxic conditions^7^-^9^. Recently, dysregulation of P4HA2 expression was found in various cancers such as oral cavity squamous cell carcinoma and breast cancer while the expression levels of P4HA2 were associated with prognosis^10^, ^11^.

Epithelial-mesenchymal transition (EMT) is a developmental process whereby epithelial cells are transcriptionally reprogrammed to a mesenchymal-like phenotype, with decreased adhesion and enhanced migration or invasion^12^. Increasing evidences suggest that collagen biosynthesis and deposition are closely involved in the EMT process in cancer progression^13^, ^14^. EMT is considered a common trait of multiple malignancies and partially responsible for the highly metastatic behaviour and aggressive nature of cancers^15^, ^16^. Promising evidence indicates that the EMT process greatly contributes to the progression of a majority of cancers by promoting invasion and metastasis, including cervical cancer^17^. However, the underlying mechanisms by which the EMT processes are regulated in cervical cancer have not been fully elucidated.

In this study, we found that P4HA2 was markedly upregulated in cervical cancer and high P4HA2 expression predicted poor clinical outcomes based on tissue microarray analysis. Consistent results were observed in a cervical cancer cohort downloaded from The Cancer Genome Atlas (TCGA). Functional analysis showed that P4HA2-silenced cervical cancer cells exhibited significantly suppressed proliferative and metastatic capacities. Furthermore, P4HA2 knockdown inhibited cervical cancer tumor xenograft growth in nude mice. Mechanistically, we revealed the positive association between highP4HA2 expression and activation of the EMT process, indicating that P4HA2 might play an oncogenic role in cervical cancer progression by inducing EMT. Taken together, our results suggest that P4HA2 functions as an oncogene in promoting cervical cancer cell proliferation, migration and invasion by inducing EMT, which might be a promising prognostic factor and therapeutic target for cervical cancer.

## Materials and Methods

### Patients and sample collection

Primary tumors from 90 patients harbouring clinically confirmed cervical cancer were prospectively collected after diagnosis at the First Affiliated Hospital of Zhengzhou University from August 2012 to January 2014. Inclusion criteria: 1) patients were pathologically diagnosed as cervical cancer and treatment for the first time; 2) patients completed whole treatment and follow-up in our hospital; 3) patients completed and have complete follow-up data; 4) patients willing to participate. Exclusion criteria: 1) patients received treatment before admission in other hospital; 2) patients transferred to other hospitals during treatment. In 40 cases, matched non-neoplastic counterparts were collected included in the present study. Relevant clinical follow-up records were collected. Informed consent was obtained from all participants. Another TMA containing 126cervical cancer specimens and 42 paired non-tumor specimens (Outdo cervical cancer cohort, HUteS168Su01) was purchased from Outdo Biotech (Shanghai, China). The study protocols were approved by the hospital's research ethics committee and were conducted in accordance with the principles expressed in the Declaration of Helsinki.

### Bioinformatics analysis

mRNA expression profiles and corresponding follow-up clinical information of cervical cancer were obtained from the public database TCGA. The TCGA cervical cancer cohort (hereafter referred to as TCGA-CESC cohort) dataset consisted of 304 cervical cancer patients. The expression of mRNAs was normalized to Log^2^ counts and then analysed by BRB-array tools as described previously^18^.

### Total RNA extraction and quantitative real-time PCR

Total RNA was extracted from clinical samples and cell lines using TRIzol® reagent (Invitrogen, USA) and then reverse transcribed to cDNA using reverse transcription kits (Roche, Germany) following the manufacturer's recommendations. The total RNA concentration was measured using a NanoDrop spectrophotometer. Quantitative real-time PCR was carried out on Applied Biosystems 7300 real-time PCR system (Applied Biosystems, USA) as described previously^19^. GAPDH was used as internal control, and the relative expression values were normalized to GAPDH levels using Ct method (2^-△△Ct^).

### Cell lines and cell culture

The human cervical cancer cell lines CaSki, SiHa, TH-3, C33A and HeLa were obtained from ATCC-American Type Culture and maintained in our laboratory. The normal cervical epithelium cell line HeCat was obtained from the Cell Bank of the Shanghai Institute of Cell Biology. All cell lines were cultured in RPMI-1640 or DMEM medium (Keygentec, China) supplemented with 10% fetal calf serum (Clark, USA) and 100 μg/ml streptomycin sulfate in humidified atmosphere with 5% CO_2_ at 37°C.

### Cell invasion and migration assay

Migration and invasion assays were conducted as previously described using the Transwell system (Corning, USA)^20^. The cell invasion assay was performed using Matrigel-coated membranes (BD, USA) in 24-well Transwell invasion chambers. Briefly, 3 × 10^4^ cells in serum-free medium were added to the upper chambers, and medium with 10% FBS was added to the lower chamber. After 36 h incubation, cells that invaded through the membrane filter were fixed and stained with 0.5% crystal violet and then counted with a microscope. A transwell chamber without Matrigel coating was used to determine the cell migration. The cell migration assay was performed following the same procedure as the invasion assay.

### 5-Ethynyl-20-deoxyuridine (EdU) assay

Cells were trypsinzed and seeded at equal numbers into 24-well tissue culture plates, After 24 h of incubation, the DNA synthesis rate of cervical cancer was determined with the EdU assay kit (Ribobio, Guangzhou, China) according to the manufacturer's instructions. The number of EdU-positive cells were counted in three random images per well by microscopy using a 100 objective (Olympus, Tokyo, Japan).

### Cell transfection

ShRNAs targeting P4HA2 and a negative control were designed and provided by GenePharma (Shanghai, China). Transfection was conducted using LipoFiter (Hanbio, China) according the manufacturer's recommended protocol. Lentiviruses for stable knockdown of P4HA2 or the negative control construct were purchased from GenePharma (Shanghai, China).

### Western blot analysis

Western blot analysis was performed as previously described^21^. Quantification analysis of western blot was conducted with the software ImageJ (Bethesda, USA). Primary antibodies against P4HA2 (1:500, Proteintech), E-cadherin (1:800, Proteintech), N-cadherin (1:800, Proteintech), and Vimentin (1:800, Proteintech) were purchased from Proteintech (Wuhan, China). An antibody against GAPDH (1:2000, Proteintech) was used as an internal control.

### Immunohistochemistry (IHC)

The immunohistochemical staining of paraffin-embedded tissue sections was processed as previously described^22^. The immunostaining results were graded as follows: 0, no staining; 1, weak staining; 2, moderate staining; and 3, strong staining. Primary antibodies against P4HA2 (1:100, Proteintech), E-cadherin (1:200, Proteintech), N-cadherin (1:200, Proteintech), and Vimentin (1:200, Proteintech) were purchased from Proteintech (Wuhan, China). For P4HA2 IHC analysis in tissue microarray (TMA), P4HA2 staining was graded as negative (score 1+), weak (score 2+), moderate (score 3+) or strong (score 4+) for further nonparametric testing according to the percentage of cells staining positive and the staining intensity.

### *In vivo* tumorigenicity

BALB/c nude mice (4-5 weeks) were purchased from Vital River Laboratory (Beijing, China). The experiments were performed in accordance with the NIH Animal Use Guidelines and approved by the Experimental Animal Ethics Committee of The First Affiliated Hospital of Zhengzhou University as previously described^23^. For the xenograft model, 5 × 10^6^ of the indicated cervical cancer cells were subcutaneously implanted into the flanks of nude mice. All mice were grouped into (1) the P4HA2 silencing group (injected with P4HA2 stable knockdown cervical cell lines) and (2) the control group (injected with negative control cells). *In vivo* tumorigenesis assays were performed with a bioluminescence imaging system (Caliper, USA) every week. Volume was calculated as length×width^2^×1/2.

### Statistics

All statistical analyses were conducted using SPSS version 23.0 software and GraphPad Prism 6 software. All *in vitro* and *in vivo* experimental results were expressed as the mean ± standard deviation (SD) of at least three independent experiments and analysed with Student's t-test (two-tailed) and the Mann-Whitney test where necessary. Differences in clinicopathological factors between the P4HA2 high- or low-expression groups were analysed via the Chi-square test. The Kaplan-Meier and log-rank test methods were used to determine the survival rate. *P* values < 0.05 were considered statistically significant.

## Results

### P4HA2 is upregulated in cervical cancer and P4HA2 overexpression correlates with poor prognosis of cervical cancer patients

**Figure 1:**
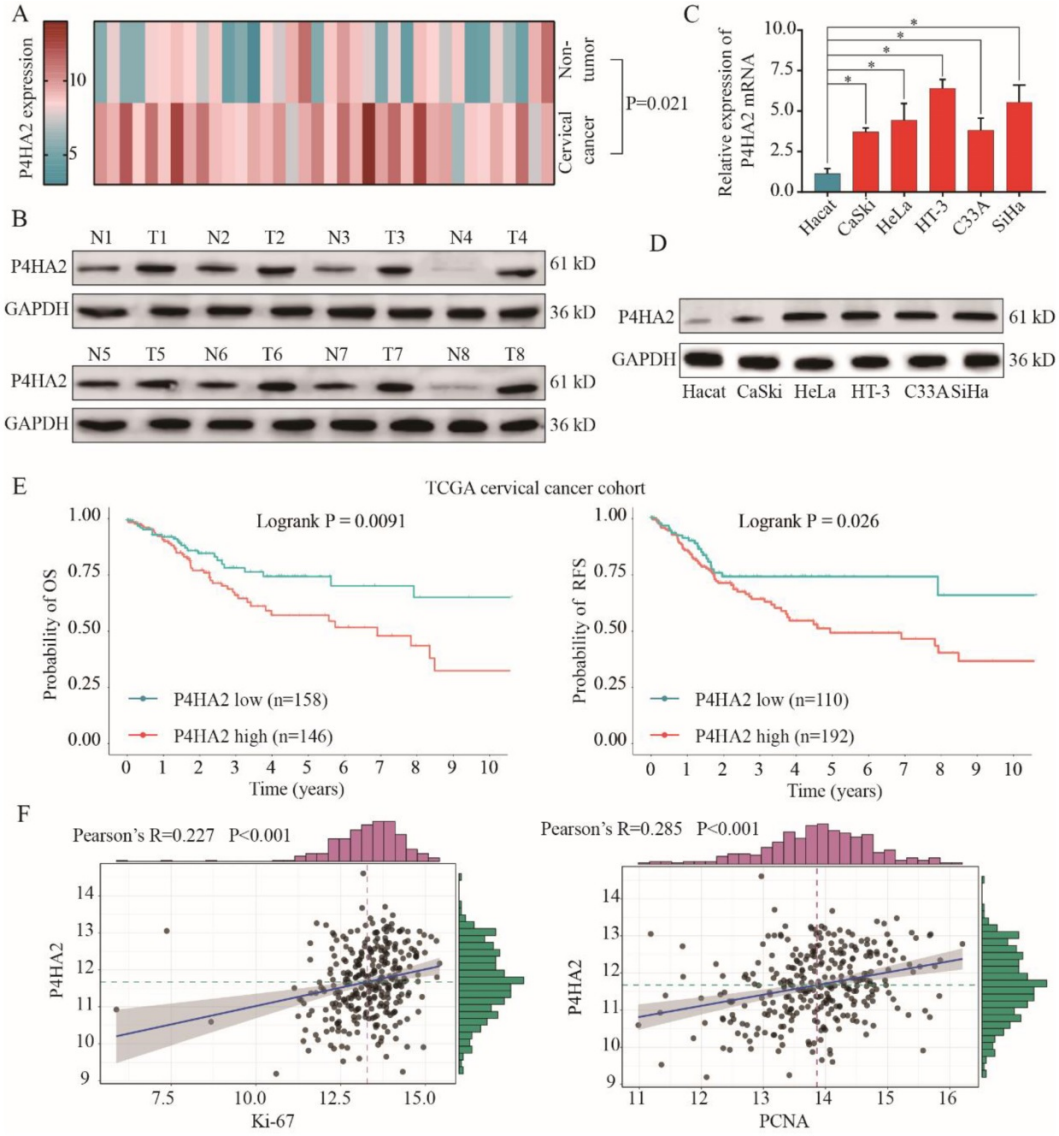
** P4HA2 is upregulated in cervical cancer and P4HA2 overexpression correlates with poor prognosis of cervical cancer patients.** (A) The expression levels of P4HA2 mRNA in 36 paired cervical cancer tissues and adjacent non-tumor tissues were analyzed by qRT-PCR. (B) The expression levels of P4HA2 mRNA in normal cervical epithelium cell line (Hecat) and five cervical cancer lines (CaSKi, HeLa, HT-3, C33A, and SiHa) were analyzed by qRT-PCR. (C)Western blots were performed to examine the expression levels of the P4HA2 protein in 8 paired cervical cancer and adjacent non-tumor tissues. GAPDH was used as an internal loading control. (D) Western blots were performed to examine the expression levels of the P4HA2 protein in normal cervical epithelium cell and cervical cancer lines. (E)Kaplan-Meier analysis of the overall survival (OS) and relapse-free survival (RFS) of patients with high or low P4HA2 expression in TCGA cervical cancer cohort. (F) Pearson correlation analysis between the expression of P4HA2 and proliferation marker Ki-67 or PCNA. The mRNA levels of above genes were acquired from the TCGA cervical cancer dataset. Results were shown as mean ± SD. *p< 0.05.

We first investigated the expression levels of P4HA2 in 36 pairs of cervical cancer tissues and matched non-neoplastic counterparts. As shown in **Figure 1A**, qRT-PCR results showed that P4HA2 was significantly upregulated in cervical cancer tissues compared with adjacent non-tumor tissues. We also found that P4HA2 expression was significantly higher in multiple cervical cancer cells (CaSKi, HeLa, HT-3, C33A and SiHa) in comparison with that in normal cervical epithelium cell line HeCat(**Figure 1B**). Consistently, western blot results further demonstrated the higher expression levels of P4HA2 protein in pairs cervical cancer tissues and cervical cancer cell lines compared with those in matched non-neoplastic tissues and normal control cell line (**Figure 1C** and **1D**).

To further evaluate the role of P4HA2 in cervical cancer, we downloaded the high throughput RNA-sequencing data of 304 cervical cancer patients for whom overall survival (OS) and relapse-free survival (RFS) follow-up data were available were from TCGA dataset (https://tcga-data.nci.nih.gov/tcga/). The correlation between P4HA2 mRNA expression levels and clinical outcomes were examined through Kaplan-Meier analysis (**Figure 1E**). We found that cervical cancer patients with high P4HA2 expression had significant lower probabilities of OS and RFS in comparison with patients with low P4HA2 expression (**Figure 1E**). Moreover, P4HA2 expression was positively related to the expression of Ki67 and PCNA, two neoplasm proliferation markers (**Figure 1F**).

**Figure 2:**
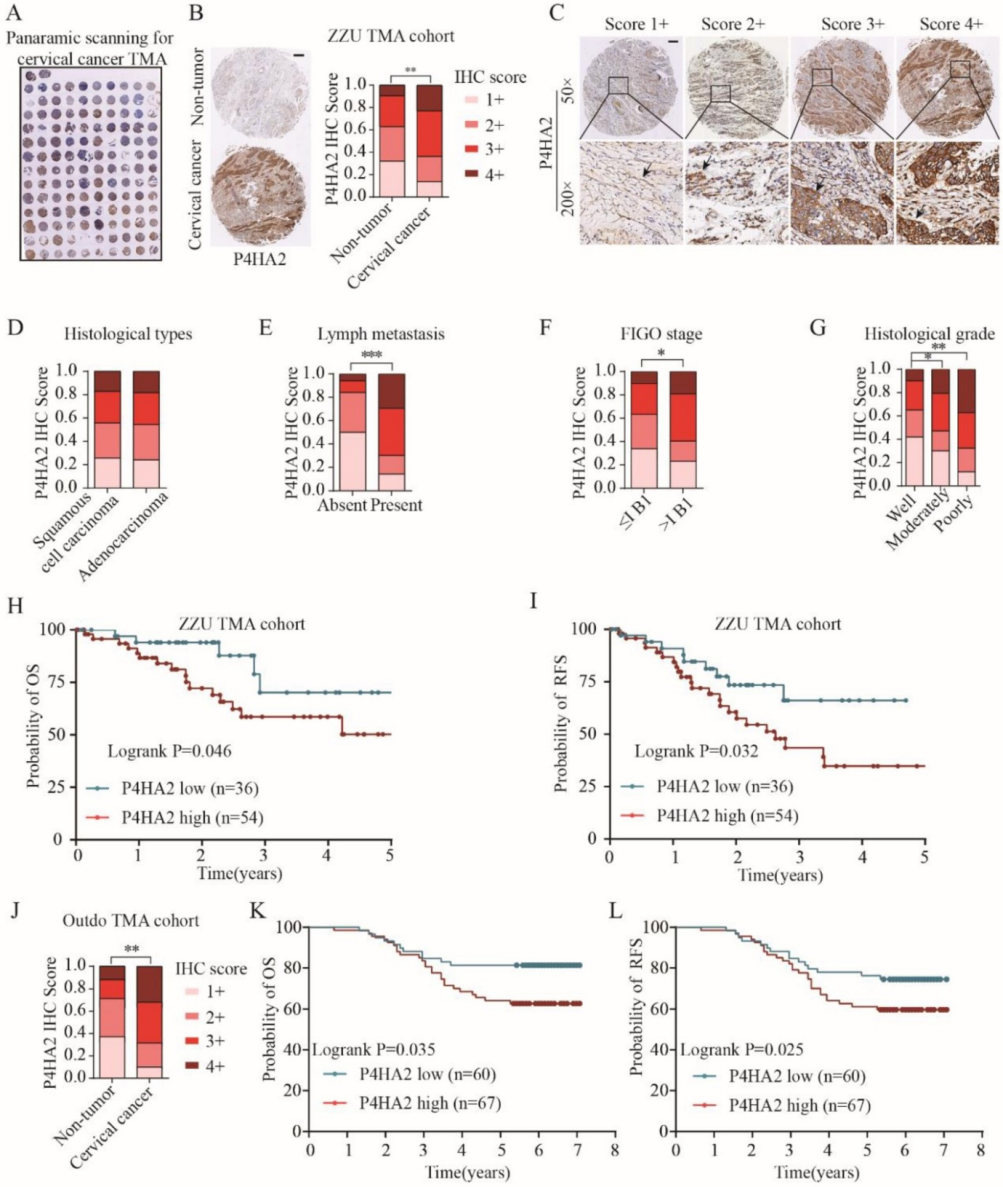
** Upregulated P4HA2 expression is associated with poor prognosis in ZZU cervical cancer TMA cohort.** (A) Panoramic scanning for cervical cancer tissue microarray (TMA) containing 90 cervical cancer tissues and 40 non-tumor adjacent tissues. (B) Representative P4HA2 IHC staining patterns in cervical cancer tissue and normal non-tumor tissues. Scale bar, 200 μm. (C) Histological scoring of P4HA2 in cervical cancer tissues. Black arrows showing the positive P4HA2 staining cells (D-G) Distribution of P4HA2 IHC staining scores in cervical cancer tissues with different histological types (D), lymph metastasis (E), FIGO stage (F), and histological grade (G). (H) The probability of OS and (I) RFS were analyzed by Kaplan-Meier analysis, according to P4HA2 staining scores in ZZU TMA cohort. (J)Distribution of P4HA2 IHC staining scores in cervical cancer tissues and non-tumor tissues in Outdo TMA cohort. (K) The probability of OS and (L) RFS were analyzed by Kaplan-Meier analysis, according to P4HA2 staining scores in Outdo TMA cohort. Results were shown as mean ± SD. *p< 0.05, **p< 0.01, and ***p< 0.001.

Upregulation of P4HA2 expression in cervical cancer was further confirmed by IHC staining on a tissue microarray (TMA) containing 90 cervical cancer tissues and 40 non-tumor adjacent tissues (**Figure 2A**) (**Table 1, ZZU TMA cohort**). Consistent with the enhanced mRNA levels in cervical cancer tissues (**Figure 1A**), IHC staining showed P4HA4 protein expression was significantly increased in the cervical cancer tissues compared with that in normal tissues (**Figure 2B and 2C**). Statistical analysis showed that there was no significant difference of P4HA2 expression between cervical squamous cell carcinoma and adenocarcinoma (**Figure 2D**). However, patients with high P4HA2 expression had a higher rate of lymph node metastasis (**Figure 2E**), more advanced FIGO stage (**Figure 2F**) and poorer histological grade (**Figure 2G**). Furthermore, Kaplan-Meier analysis revealed shorter OS (*p*=0.046,** Figure 2H**) and RFS times (*p*=0.032,** Figure 2I**) in patients with high P4HA2 expression. Our data was further validated using another commercial TMA cohort (Outdo cervical cancer cohort, HUteS168Su01) (**Figure 2J, 2K and 2L**).

We also performed univariate analysis of prognostic factors for OS with Cox's regression model in the ZZU cervical cancer cohort (**Table 2**). Lymph node metastasis (*p*=0.002, HR=2.388), poor differentiation (*p*=0.038, HR=1.863), advanced FIGO stage (*p*=0.019, HR=2.501) and high P4HA2 expression (*p*=0.024, HR=2.709) were significantly associated with poor survival. Of note, multivariate analysis indicated that high expression levels of P4HA2 *p*=0.037, HR=1.825) were significantly associated with worse prognosis in cervical cancer patients independently of advanced FIGO stage (*p*=0.024, HR=2.307) and lymph node metastasis (*p*=0.000, HR=2.611). The results suggest that P4HA2 is an independent prognostic factor and that high P4HA2 expression is associated with poorer survival in cervical cancer.

### P4HA2 knockdown suppresses cervical cancer cell proliferation, migration and invasion *in vitro*

**Figure 3:**
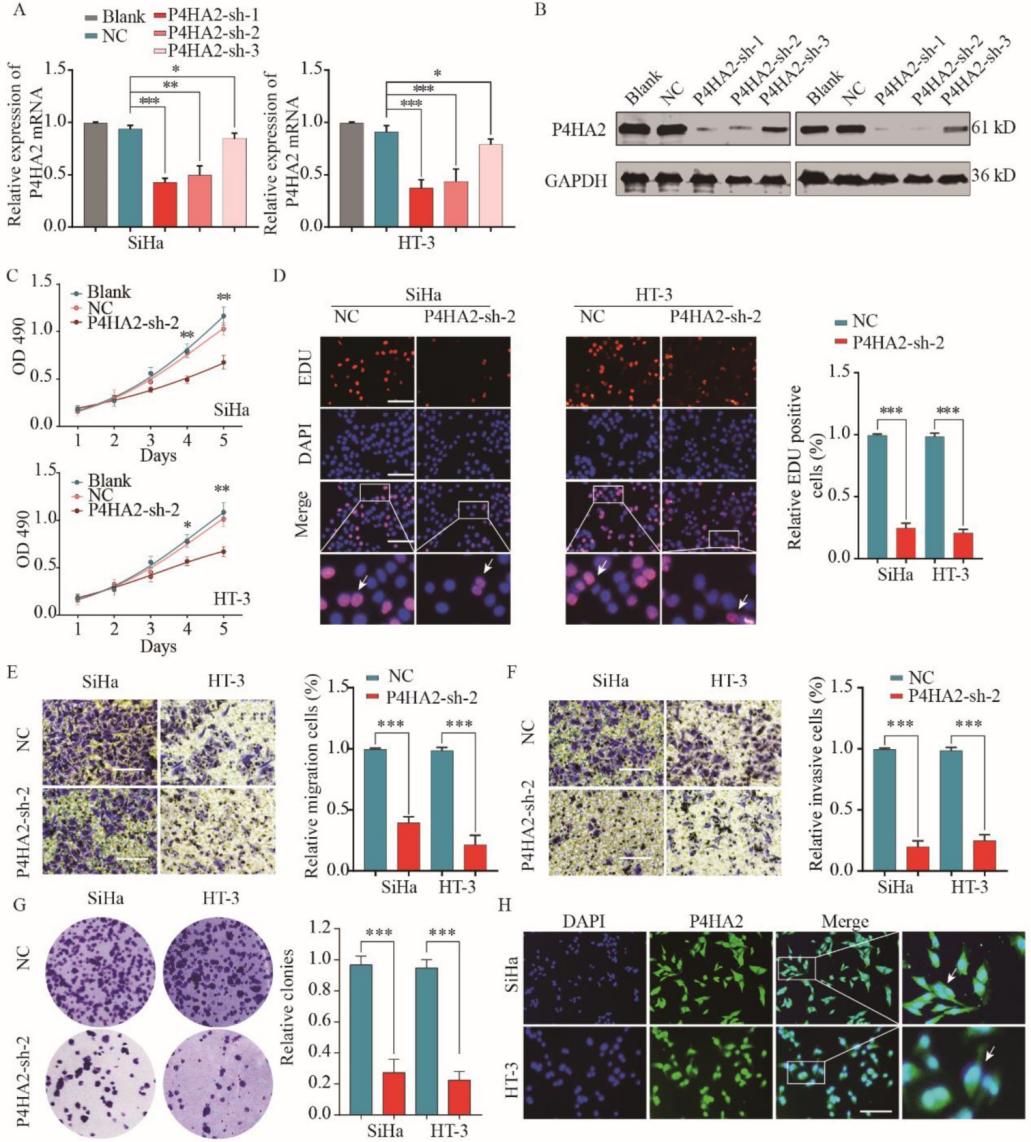
** P4HA2 knockdown suppresses cervical cancer cell proliferation, migration and invasion *in vitro*.** Cervical cancer SiHa or HT-3 cells were transfected with shRNAs targeting P4HA2 (P4HA2-sh-1/2/3) or negative control (NC). Knockdown efficiency was determined by qRT-PCR (A) and western blot (B) respectively. (C) Cell proliferation of SiHa or HT-3 was assessed by CCK-8 assay. (D) Cell proliferation of SiHa or HT-3 cells was assessed by EDU/DAPI staining. Scale bar, 100 μm. White arrows showing the positive EdU staining cells. (E) Cell migration and (F) invasion of SiHa or HT-3 cells were analyzed by transwell assay. (G) The colony formation of SiHa or HT-3 transfected with P4HA2-sh-2 or NC was analyzed. (H) Immunofluorescence analysis of P4HA2 expression in cervical cancer cell lines SiHa and HT-3. White arrows showing the positive IF staining cells. Results were shown as mean ± SD. *p< 0.05, **p< 0.01, and ***p< 0.001.

To further explore the function of P4HA2 in cervical cancer progression, we evaluated three different shRNAs targeting P4HA2 (P4HA2-shRNA-1/2/3) in SiHa and HT-3 cells, which had the highest levels of P4HA2. The knockdown efficiency was determined by qRT-PCR and western blot (**Figure 3A** and **3B**). P4HA2-sh-2 had the highest silencing efficiency and was used for the subsequent experiments. The CCK-8 assay showed that P4HA2 knockdown significantly inhibited cell proliferation in SiHa and HT-3 cells (**Figure 3C**). Consistently, we observed a remarkable decrease in cell proliferation after P4HA2 silencing compared with that in the control group using EDU/DAPI staining assays (**Figure 3D**). Furthermore, Transwell migration assays showed that the relative migration cells of cervical cancer cells were remarkably decreased after P4HA2 knockdown (**Figure 3E**). Matrigel invasion assay also demonstrated that knockdown P4HA2 inhibited the invasive activities of SiHa and HT-3 cells (**Figure 3F**). In addition, we demonstrated that P4HA2 knockdown suppressed the capability of colony formation in SiHa and HT-3 cells (**Figure 3G**). As shown in **Figure 3H**, P4HA2 were mostly distributed in cell cytoplasm of cervical cancer cells.

### P4HA2 knockdown suppresses cervical cancer tumorigenesis *in vivo*

**Figure 4:**
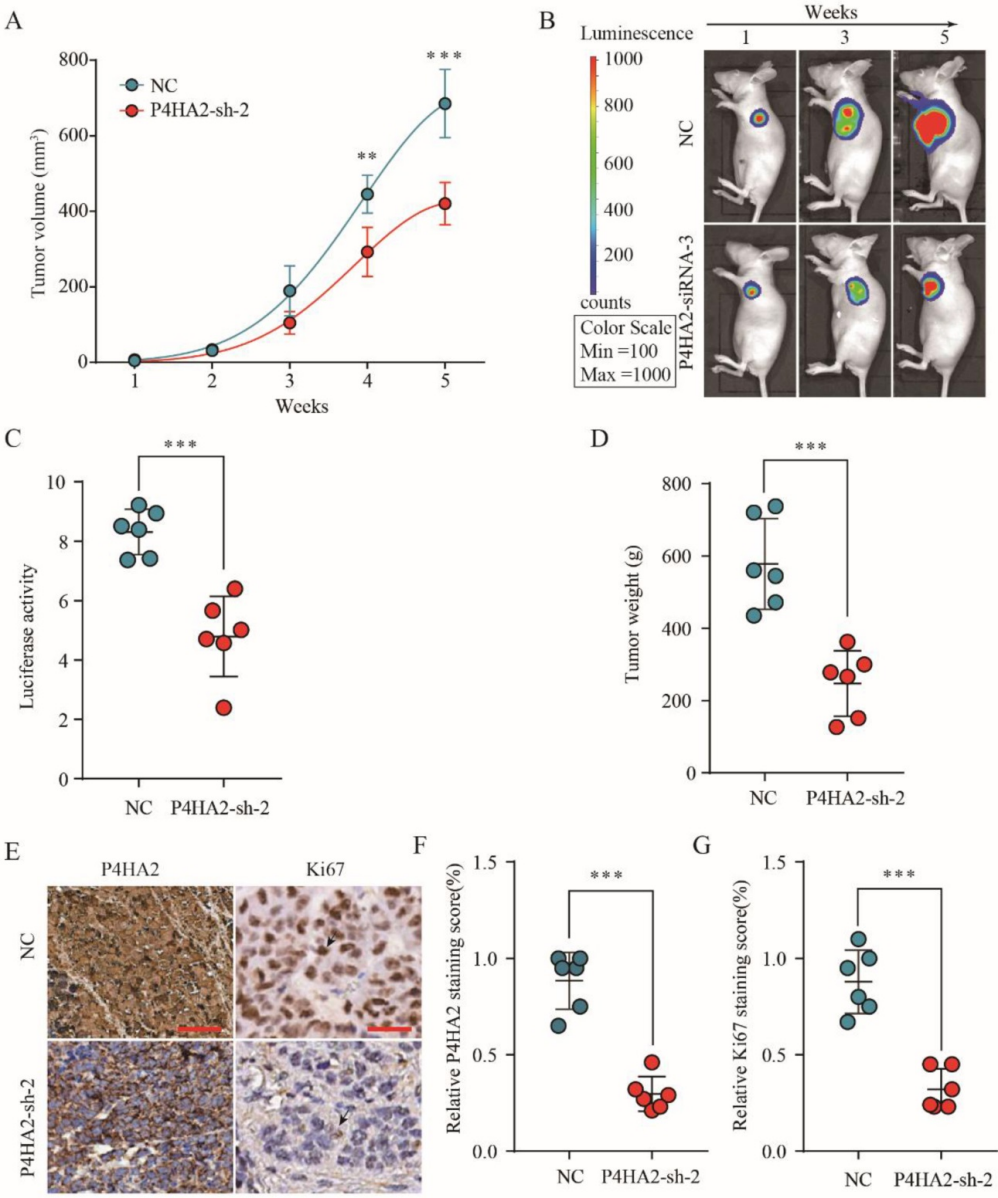
** P4HA2 knockdown suppresses cervical cancer tumorigenesis *in vivo.*** (A) Growth curves of tumor volumes in xenografts of nude mice were determined based on tumor volume measured every week for 5 weeks. (B, C) Photographs of tumor formation in nude mice were taken by a live imaging system detecting the luciferase signal, and the luciferase signal in the P4HA2 knockdown group and control group were analyzed 5 weeks after implantation. (D) Tumor weights of P4HA2 knockdown group and control group were analyzed at 5 weeks. (E-G) Xenograft tumors tissues from P4HA2 knockdown group or control group were stained for P4HA2 and Ki67. The relative P4HA2 and Ki67 staining scores were analyzed. Scale bar, 20 μm. Black arrows showing the positive IHC staining cells. Representative images and data are based on three independent experiments. **p< 0.01 and ***p< 0.001.

To investigate the effect of P4HA2 knockdown on cervical cancer tumorigenesis, we established a SiHa cell line with stable knockdown of P4HA2. SiHa cells with stable knockdown of P4HA2 were subcutaneously implanted into nude mice, and tumor formation and growth were monitored. As expected, P4HA2 knockdown drastically inhibited the growth of tumors (**Figure 4A**). Bioluminescence imaging revealed that the tumors in the mice implanted P4HA2-silenced SiHa cells had a significant decreased mean luciferase signal compared with tumors developed in the control group (**Figure 4B** and **4C**). Consistently, the weight of the tumors enucleated from the P4HA2 knockdown group was significantly lower in comparison with that in control group (**Figure 4D**). IHC staining showed that the relative P4HA2 staining was remarkably weaker in the tumor tissues from P4HA2 knockdown group (**Figure 4E** and **4F**). Moreover, we also detected decreased Ki67 expression in the tumor tissues obtained from P4HA2 knockdown group mice compared with those from the negative control group (**Figure 4E** and** 4G**). Collectively, these results demonstrated that P4HA2 knockdown was capable to suppress the tumorigenesis of cervical cancer* in vivo*.

### Bioinformatics analysis of TCGA cervical cancer cohort with high or low P4HA2 expression

**Figure 5:**
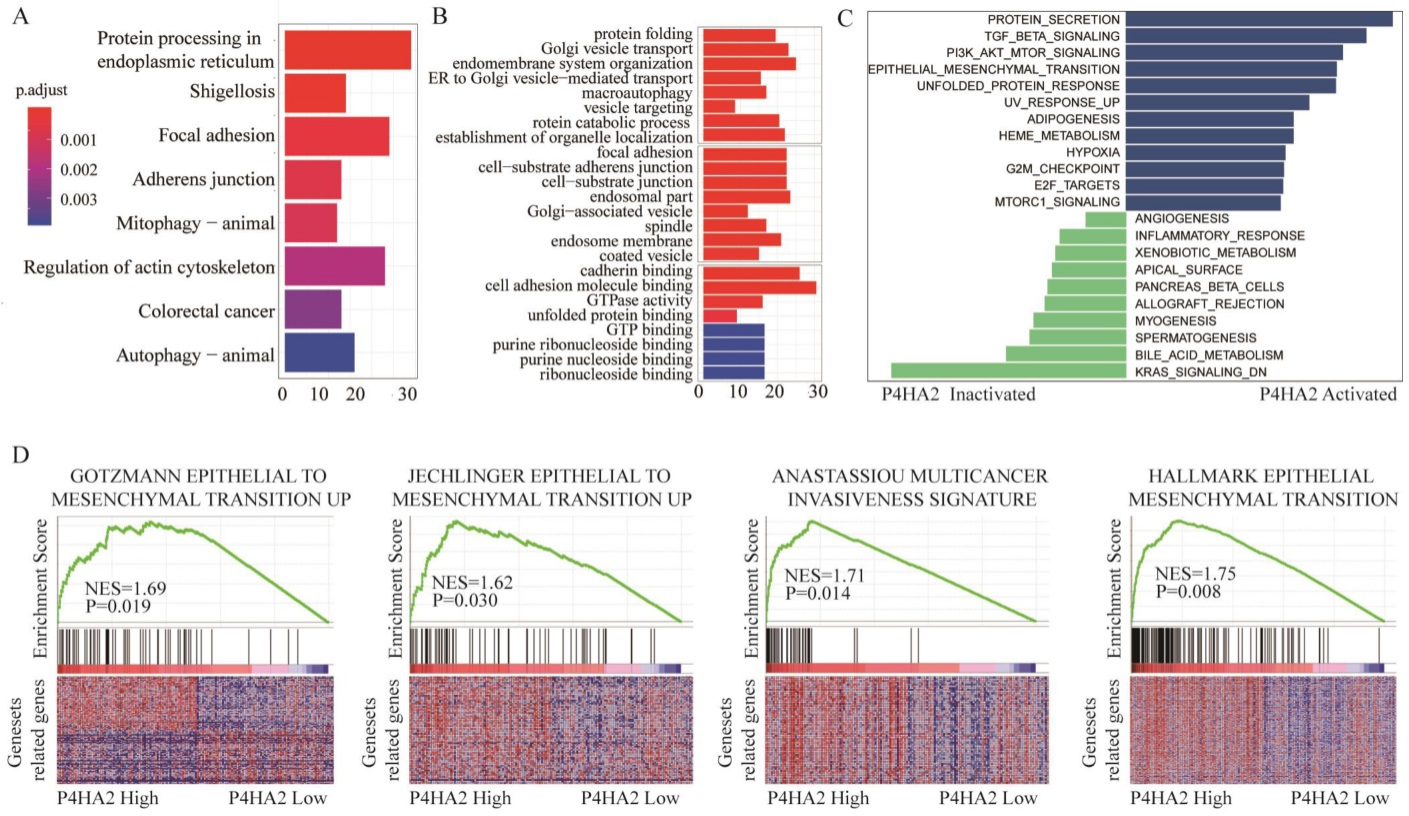
** Bioinformatics analysis of TCGA cervical cancer cohort with high or low P4HA2 expression.** (A) KEGG pathway enrichment analysis and (B) GO enrichment analysis of the top 800 genes with highest P4HA2 correlation coefficient in TCGA cervical cancer cohort.(C) Gene set variation analysis (GSVA) and (D)The Gene Set Enrichment Analysis (GSEA) of the relationship between the expression level of P4HA2 and EMT-related gene signatures in the TCGA cervical cancer dataset.

To explore the underlying mechanisms by which P4HA2 knockdown inhibits tumor growth of cervical cancer, we next conducted KEGG pathway enrichment analysis (**Figure 5A**) and Gene Ontology (GO) enrichment analysis (**Figure 5B**) of the top 800 genes with the highest P4HA2 correlation coefficients in TCGA cervical cancer cohorts. We found that the biological processes including “Focal adhesion” and “Adherens junction” were enhanced in cervical cancer patients with high P4HA2 expression (**Figure 5A**). The GO enrichment analysis also showed enhanced activities including “focal adhesion”, “cell-substrate adherens junction”, “cadherin binding” and “cell adhesion molecule binding” in cervical cancer patients with high P4HA2 expression (**Figure 5B**). Numerous studies have documented that these biological processes and pathways are closely involved in the EMT process, which is a well-recognized biological process associated with tumor metastasis^24^, ^25^. Thus, we analyzed relationship between the expression level of P4HA2 and EMT-related gene signatures via Gene set variation analysis (GSVA) (**Figure 5C**) and Gene Set Enrichment Analysis (GSEA) (**Figure 5D**). The results validated the enhanced activity of Gotzmann epithelial to mesenchymal transition up, Jechlinger epithelial to mesenchymal transition up, and Anastassiou multicancer invasiveness signature in patients with high levels of P4HA2 (**Figure 5D**).

### P4HA2 promotes cervical cancer progression and metastasis via inducing EMT

**Figure 6:**
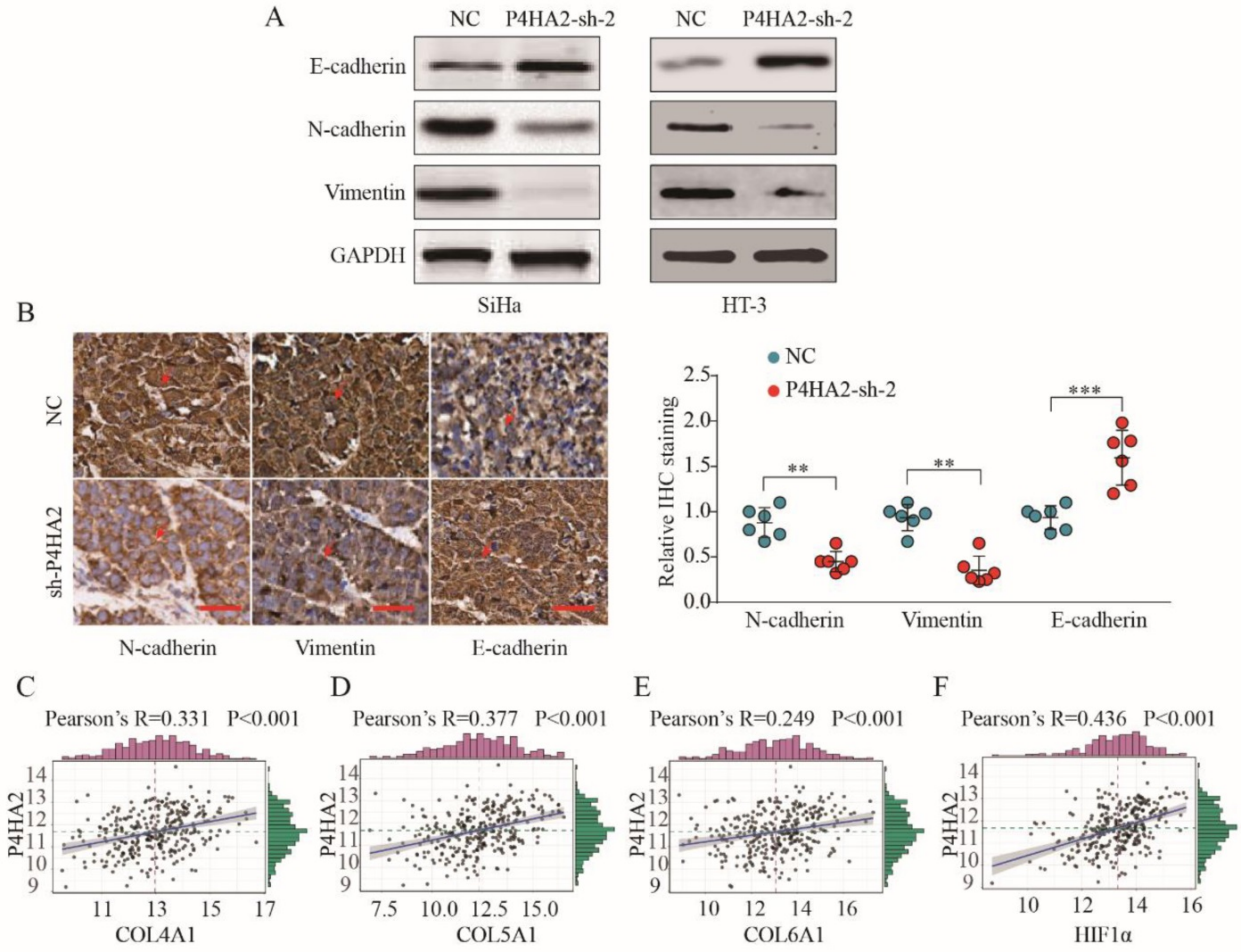
** P4HA2 promotes cervical cancer progression and metastasis via inducing EMT.** (A) Western blot analysis of E-cadherin, N-cadherin and Vimentin in SiHa or HT-3 cells transfected with P4HA2-sh-2 or negative control. (B) IHC staining of N-cadherin, Vimentin, and E-cadherin in tumor tissues from P4HA2 silencing group or the negative control group. Scale bar, 20 μm. Red arrows showing the positive IHC staining cells. **p< 0.01 and ***p< 0.001. Unpaired Student's *t*-test. Representative images and data are based on three independent experiments. (C-F) Pearson correlation analysis between the expression of P4HA2 and (C) Col4A1, (D) Col5A1, (E) Col6A1, or (F) HIF-1α in cervical cancer tissues (n = 307). The mRNA levels of above genes were from the TCGA dataset.

We subsequently detected the expression levels of EMT process-related markers in cervical cancer cells transfected with P4HA2-sh-2 or negative control. The results showed that Vimentin and N-cadherin expression were significantly decreased while E-cadherin levels were increased in P4HA2-silenced SiHa cells (**Figure 6A**). Consistent with *in vitro* data, IHC staining showed weaker Vimentin and N-cadherin expression but stronger E-cadherin expression in tumor tissues from the P4HA2-silenced group than in those from the control group (**Figure 6B**). Previous report demonstrated that hypoxia could promote the cancer invasion process by regulating collagen biosynthesis and deposition^26^. We further investigated the relationship between P4HA2 expression and the expression of collagen related genes and hypoxia-inducible factor (HIF)-1α. As shown in **Figure 6C-F**, the expression levels of Col1A1, Col3A1, Col4A1 and HIF-1α were positively correlated with P4HA2 levels in TCGA cervical cancer cohort (P < 0.001). Collectively, these data suggest that silencing P4HA2 can attenuate the aggressive activities of cervical cancer cells, at least in part, by regulating the EMT process.

## Discussion

EMT has been demonstrated to be involved in metastasis of primary tumors including cervical cancer^27^. In addition, emerging evidences have revealed that collagen biosynthesis and deposition are closely involved in the EMT process in cancer progression^13^, ^14^. In the current study, we demonstrated collagen P4HA2 was upregulated in cervical cancer and P4HA2 overexpression correlated with poor prognosis of cervical cancer patients. Using both *in vitro* cell line and *in vivo* xenograft tumor model, we found knockdown P4HA2 inhibited cervical cancer development and metastasis. Mechanistically, bioinformatics analysis indicated EMT process was involved and we further validated that P4HA2 promoted cervical cancer progression and metastasis via inducing EMT.

P4HA2 is a key protein in collagen biosynthesis and plays a crucial role in promoting various tumor progressions. Chang et al. demonstrated a close connection between upregulated P4HA2 expression and metastatic potential in oral cavity squamous cell carcinoma^10^. In breast cancer, Xiong et al. observed that P4HA2 could promote breast cancer metastasis by inducing collagen deposition^11^. Consistent with these findings, our study confirmed that the significant upregulation of P4HA2 in cervical cancer was correlated with metastasis and advanced FIGO stage (**Figure 1** and **2**). These concordant results emphasize the critical role of P4HA2 in cancer progression. In addition, we demonstrated that knockdown P4HA2 inhibited the proliferation, migration and invasion of cervical cancer cells *in vitro* (**Figure 3**) and suppressed cervical tumor growth *in vivo* (**Figure 4**).

Bioinformatics analysis indicated that EMT-related mechanisms underlined the functional role of P4HA2 in cervical cancer (**Figure 5**), we hypothesized that P4HA2 could promote cervical cancer cell invasion by regulating the EMT process. Consistent with this hypothesis, we observed that knockdown of P4HA2 suppressed the protein levels of downstream effectors of EMT (N-cadherin and Vimentin) both *in vitro and in vivo*, supporting a metastasis-promoting role of P4HA2. Collagen prolyl-4-hydroxylase has been identified as a marker of collagen synthesis that is essential for collagen stabilization^28^. Our data revealed a close positive correlation between the expression of P4HA2 and collagen biosynthesis-related genes (Col4A1, Col5A1 and Col6A1) in cervical cancer (**Figure 6**), which is also consistent with a previous report of a positive correlation between collagen biosynthesis and P4HA2 in breast cancer^11^. These results indicate that P4HA2 may be involved in the metastatic process in cervical cancer by regulating collagen biosynthesis. Thus, P4HA2 might be a promising therapeutic target for cervical cancer patients.

A recent study documented that hypoxia-inducible factor 1 can enhance P4HA2 expression to subsequently promote collagen fibre alignment^8^. Tumor hypoxia could promote invasion and metastasis by inducing EMT process in various cancers^29^-^31^. We also observed a significant correlation between P4HA2 and HIF1a expression in our study and speculated that hypoxia might regulate the EMT process by enhancing the expression of P4HA2^32^, ^33^. Taken together, the data suggest that P4HA2 might serves as an oncogene via activating the EMT process in cervical cancer. The molecular mechanisms underlying the promoting effect of P4HA2 require further experimentation.

To our knowledge, this is the first report to investigate the biological function of P4HA2 in cervical cancer cells *in vitro* and we have validated the relationship between P4HA2 expression and the prognosis of cervical cancer patients by using TCGA datasets and two sets of cervical cancer tissue microarray. In addition, we also demonstrated that P4HA2 knockdown suppressed EMT process and tumor metastasis.

## Conclusion

In this study, we showed that P4HA2 is highly expressed in cervical cancer and that upregulation of P4HA2 is correlated with aggressive phenotypes and poor prognosis. Functionally, silencing P4HA2 suppresses the proliferation and metastatic ability of cervical cancer *in vitro* and* in vivo*. Mechanistically, P4HA2 plays an oncogenic role by promoting the EMT process in cervical cancer. Taken together, P4HA2 might serve as a novel prognostic biomarker and promising therapeutic target in cervical cancer.

## Figures and Tables

**Table 1:** Correlation of clinico-pathological features with P4HA2 expression in cervical cancer ZZU TMA cohort

Variables	Clinicopathological features	Total (n=90)	P4HA2 expression level		*P- value*
			Low (n, %)	high (n, %)	
Age (years)	<45	32	12 (33.3)	20 (45.4)	0.719
	>45	58	24 (66.7)	34 (54.5)	
Histologic subtype	Squamous cell carcinoma	58	22 (61.1)	36 (66.7)	0.589
	Adenocarcinoma	32	14 (38.9)	18 (33.3)	
FIGO stage	I	53	26 (72.2)	27 (50.0)	0.035
	II	37	10 (27.8)	27 (50.0)	
Lymph metastasis	No	59	29 (80.5)	30 (55.5)	0.014
	Yes	31	7 (19.4)	24 (44.4)	
Differentiation grade	Well & Moderate	55	27 (75.0)	28 (19.4)	0.027
	Poor	35	9 (25.0)	26 (19.4)	
Carcinoma diameter (cm)	<4	61	26 (72.2)	35 (64.8)	0.461
	>4	29	10 (27.8)	19 (35.1)	
Vascular involvement	No	57	27 (75.0)	30 (55.5)	0.061
	Yes	33	9 (25.0)	24 (44.4)	

**Table 2:** Correlation of clinico-pathological features with P4HA2 expression in cervical cancer TMA cohort

Clinicopathological features	Univariate analysis			Multivariate analysis		
	HR	95% CI	*P* value	HR	95% CI	*P* value
Age (>45 vs. <45)	1.137	0.708-1.958	0.782			
Histologic subtype (Squamous cell carcinoma *vs.* Adenocarcinoma)	0.769	0.556-1.392	0.629			
FIGO stage (stage II *vs.* stage I)	2.501	1.823-3.482	0.019	2.307	1.784-2.981	0.024
Lymph metastasis (Yes *vs.* No)	2.388	1.739-3.180	0.002	2.611	2.058-3.438	0.000
Differentiation grade (Poor *vs.* Well & Moderate)	1.863	1.529-2.054	0.038	1.309	1.101-1.773	0.098
Carcinoma diameter (>4cm *vs.*<4cm)	1.149	1.129-1.454	0.129	1.420	0.820-1.131	0.283
Vascular involvement (Yes *vs.* No)	1.460	1.284-1.827	0.078			
P4HA2 expression (High *vs.* low)	2.079	1.451-2.941	0.024	1.825	1.604-2.321	0.037

## Abbreviations

P4HA2: prolyl-4-hydroxylase α subunit 2
OS: overall survival
RFS: Relapse-free survival
EMT: epithelial-mesenchymal transition
IHC: immunohistochemistry
EdU: 5-ethynyl-20-deoxyuridine
GSEA: Gene Set Enrichment Analysis
TMA: tissue microarray
TCGA: The Cancer Genome Atlas
GO: gene ontology
